# Breastfeeding and risk of hospitalisation in children under five years—a systematic review and meta-analysis

**DOI:** 10.3389/fped.2026.1748152

**Published:** 2026-02-09

**Authors:** Sushmita Kerketta, Pritimayee Sethy, Sasmita Swain, Satyabrata Pradhan, Sameer Sahu, Rutusmita Sahoo, Mahipal Munda, Srikanta Kanungo, Manikandan Srinivasan, Sanghamitra Pati

**Affiliations:** 1 Department of Public Health, ICMR–Regional Medical Research Centre, Bhubaneswar, India; 2 Centre for Evidence for Guidelines, Department of Health Research, New Delhi & Indian Council of Medical Research, New Delhi, India; 3 Epidemiology, JIPMER International School of Public Health, JIPMER, Pondicherry, India; 4 Indian Council of Medical Research, New Delhi, India

**Keywords:** breastfeeding (BF), duration of breast feeding, gastro-intestinal infections, hospitalization, respiratory infection, under 5 children

## Abstract

**Objective:**

To evaluate the association between breastfeeding duration and risk of diarrhoea- and respiratory infection-related, infection-related and all-cause hospitalisation in under-five children from cohort studies.

**Methods:**

This systematic review was conducted in accordance with PRISMA (Preferred Reporting Items for Systematic Reviews and Meta-Analyses) guidelines. We conducted a systematic literature search across PubMed, Embase, CINAHL, Scopus and Google Scholar for studies related to breastfeeding and hospitalisation in early childhood. Three authors independently screened titles and abstracts, followed by full texts assessment as per eligibility criteria. Data abstracted were pooled using random effects model, and estimates were presented as odds ratios. Odds of hospitalisation were reported across various exposure categories - exclusively breastfed, predominantly breastfed, partially breastfed and never breastfed under age strata of <1 year, 1–2 years and 2–5 years. The New Castle Ottawa scale for cohort studies was used for risk of bias assessment.

**Findings:**

Total 16 cohort studies, including 27,80,195 children, across global settings, were included in this systematic review, and the majority (13, 81.25%) of studies were performed in high-income country settings. Children under one year of age who were exclusively breastfed had an insignificant reduction in odds of hospitalisation due to gastrointestinal infections compared to those never breastfed, with pooled OR of 0.79 (0.57–1.09), I2–53%. In the case of respiratory infection-specific hospitalisation, a significant reduction in odds of hospitalisation was noted in children across the age bands of <1, 1–2 and 2–5 years with pooled ORs of 0.88 (0.82–0.95), I2–96%; 0.82 (0.70–0.97), I2–61% and 0.73 (0.56–0.95), I2 – NA, respectively. Children <1 year with EBF significantly reduced odds of all-cause hospitalisation compared to those never breastfed with pooled OR of 0.97 (0.97–0.97), I2 – NA.

**Conclusion:**

This meta-analysis estimated that exclusive breastfeeding practice in children is associated with a significant reduction in odds of hospitalisation due to respiratory infections, and not in the case of gastrointestinal diseases, with greater benefits in the first year of life, based on studies with considerable heterogeneity.

**Systematic Review Registration:**

https://www.crd.york.ac.uk/prospero/, identifier CRD42024537665.

## 1 Introduction

The United Nations Inter-Agency Group for Child Mortality has estimated that under-five mortality has reduced from 93 deaths for 1,000 live births in 1990 to 37.7 in 2019. In 2019, low-and-middle-income countries (LMICs) shared 59% of global under-five deaths, with lower respiratory infections and diarrhoea being the leading causes of death in under-five children. Lives Saved Tool (LiST) has modelled age-appropriate breastfeeding, as recommended by the World Health Organization (WHO), comprising of exclusive breastfeeding for 6 months and continued breastfeeding until two years of age, reduced under-five mortality through a reduction in diarrhoeal incidence and stunting in children in LMICs (1). In the Indian context, evidence from a large trial showed that exclusive breastfeeding at three months reduces infant mortality by 1.6 and 11.9 times compared to partially and never breastfed infants, respectively, over three—six months of age (2). Beyond mortality reduction, optimal breastfeeding practices during early infancy have been protective against severe morbidity due to infectious causes, such as reduction in hospitalisation events for diarrhoea and acute lower respiratory tract infections. Thus, optimal breastfeeding has been implicated as an important intervention, along with immunisation and upgrading WaSH infrastructure, in the reduction of mortality and hospitalisations in children from developing settings due to infectious causes (3, 4).

A systematic review published in 2011 summarised the risk between breastfeeding practices and diarrhoeal hospitalisation and concluded that exclusively breastfed children had a risk of hospitalisation comparable to that of predominantly breastfed and, with a significant risk reduction by 77% and 95% compared to partially and never breastfed children, respectively (5). Similarly, previous meta-analyses published in 2003, 2011, and 2022 measured the risk of respiratory infection-related hospitalisation and pneumonia-specific hospitalisation, respectively, due to suboptimal breastfeeding practices in children (5–7). These systematic reviews summarised that children who never breastfed had a higher risk for hospitalisation compared to those who exclusively breastfed. Considering that the previous reviews were performed during the last decade, including fewer studies, and have included case-control studies, where ruling out reverse causality is challenging, it would warrant a systematic review of literature based on longitudinal designs. Thus, a systematic review was performed to evaluate the association between breastfeeding duration and hospitalisation due to all-cause (any hospitalization), infection-related, diarrhoea- and respiratory infection-related hospitalisation in under-five children based on cohort studies.

## 2 Methodology

The PRISMA (Preferred Reporting Items for Systematic Reviews and Meta-Analyses) 2020 guidelines were followed for this systematic review (8). The protocol for this review was registered in PROSPERO with ID CRD42024537665.

### 2.1 Eligibility criteria

The eligibility criteria for our review has been clearly depicted using the PICOS framework in following table (Table 1):

**Table 1:** Eligibility criteria (PICOS framework) for study selection.

Domain	Eligibility criteria
Population	*Inclusion criteria*: Studies including children under 5 years including those who were born as term or preterm Studies that included children regardless of their birth weight status, singleton status or the geographical location they belonged to. *Exclusion criteria*: *1.* Children born with congenital anomalies or any other premorbid conditions like HIV positive status, that increases their chances of hospitalization compared to general population.
Intervention (Exposure)	*Inclusion criteria*: Children who were exclusively/predominantly breastfed for at least 2 months, followed by breast feeding of any duration. Children exclusively/predominantly breastfed for ≥2 months, irrespective of pre-lacteal feeds given during initial days of life
Comparator	*Inclusion criteria*: Studies with internal comparison with children never breastfed or have not been exclusively or predominantly breastfed. Studies that included comparator group with children who were breastfed for relatively lesser duration
Outcomes	*Inclusion criteria*: Studies that reported outcomes such as risk/rates/odds of all-cause hospitalization in first 5 years of life in children Studies that reported infectious-specific/respiratory-specific/diarrhoea- specific hospitalization rates in first 5 years of life in children *Exclusion criteria*: Studies that did not provide hospitalization rates separately for children with or without congenital anomalies/premorbid conditions.
Studies	*Inclusion criteria*: Cohort studies, case-control and randomised controlled trials/non-randomized studies (control arm data), Cross-sectional surveys, *Exclusion criteria*: case-reports, case-series, letters, reviews including systematic reviews.

### 2.2 Study design

This review included either cohort studies or control arm data of randomised controlled trials and non-randomized studies published in peer-reviewed journals. The study design excluded case-control, cross-sectional, case reports, case series, letters, conference papers, and systematic reviews.

### 2.3 Information sources

We conducted a systematic literature search across PubMed, Embase, Cumulative Index to Nursing and Allied Health (CINAHL), Scopus and Google Scholar for studies on the association between the duration of breastfeeding and the risk of hospitalisation in children.

### 2.4 Search strategy

The search was performed using keywords and MeSH terms for “child”, “breastfeeding”, and “hospitalisation” combined with Boolean operators (AND, OR) to build search strings in each database. We conducted a comprehensive search of PubMed from its inception to 16 April 2024 (Supplementary File S1).

### 2.5 Study selection process

Rayyan software used for the de-duplication and screening of the records retrieved from the databases (9). Based on the predefined inclusion and exclusion criteria, titles and abstracts were screened, followed by full texts by independent reviewers in triplicate (SP, SR, MM, PS, SS and SS). Conflicts in the selection of articles were resolved by SK and MS.

### 2.6 Data collection process

A pilot-tested pro-forma was used to retrieve information from selected studies independently by four reviewers (PS, SP, SS and SR) in a Microsoft Excel sheet. Disagreements during data extraction were resolved through discussion between the reviewers or adjudication by the third reviewer (MS or SK). Data extraction was organised under four parts-

***Part I:* Study characteristics**: We collected study title, author details and affiliations, year of publication, as well as study location, design, sample size, dropouts, and follow-up duration.

***Part II:* Study population**: This included data on age, sex, birth weight, and exclusive breastfeeding characteristics reported in the cohort.

***Part III:* Exposure Information**: For the exposed group, we extracted information on the type of breastfeeding exposure (Exclusively breastfed (EBF-Exclusive breastfeeding means that the infant receives only breast milk, no other liquids or solids are given- not even water (10).) and Predominantly breastfed (PrBF- Predominant breastfeeding means the only milk given to the infant being human milk (no animal milk or infant formula (11).))) and the duration of each type of exposure. For the comparator group, we collected data on breastfeeding practices such as partially breastfed (PBF-Partial breastfeeding means that the infant may be given animal milk, formula or solids in addition to breast milk (12)), never breastfed (NBF) or formula fed along with the duration of these practices.

***Part- IV:* Outcome measures**: We collected data on causes of hospitalisation and the number of children hospitalised or not across breastfeeding exposure categories. We also extracted the type of effect measures reported, including relative risk, risk ratios or odds ratios, and 95% confidence intervals.

### 2.7 Quality assessment of studies

The New-Castle Ottawa scale was used to assess the quality of the included studies and classified according to AHRQ standards (13). The studies were assessed under selection, comparability, and outcomes. Two reviewers (SS and MM) independently assessed each study included.

### 2.8 Data synthesis and analysis

A total of 16 studies were included in this systematic review. Unadjusted odds ratios were computed for studies that did not report measures of association but provided the number of hospitalisation events in exposed and unexposed children. To harmonise outcome measures in the datasheet, relative risks and risk ratios were converted to odds ratios as per the cited literature (14). Random effects meta-analysis was performed using an inverse-variance weighing approach considering the substantial heterogeneity identified between the studies. Meta-analysis reported pooled odds ratios with 95% confidence intervals for each cause of hospitalisation: gastrointestinal infection, respiratory infection, all-cause hospitalisation, and any infection-specific hospitalisation. Subgroup analysis was carried out to report the odds of hospitalisation by comparing different practices and duration of breastfeeding across age groups, including <1 year, 1–2 years and 2–5 years.

#### 2.8.1 Heterogeneity assessment

It was assessed using the Chi-square test (*P* < 0.05) and *I*^2^ statistic. An *I*^2^ value exceeding 50% was considered indicative of substantial heterogeneity. Sources of heterogeneity in studies were explored based on the study characteristics, such as the age of participants and the duration or type of breastfeeding. Statistical analysis was carried out using Cochrane Review Manager (RevMan) software.

## 3 Results

**Figure 1:**
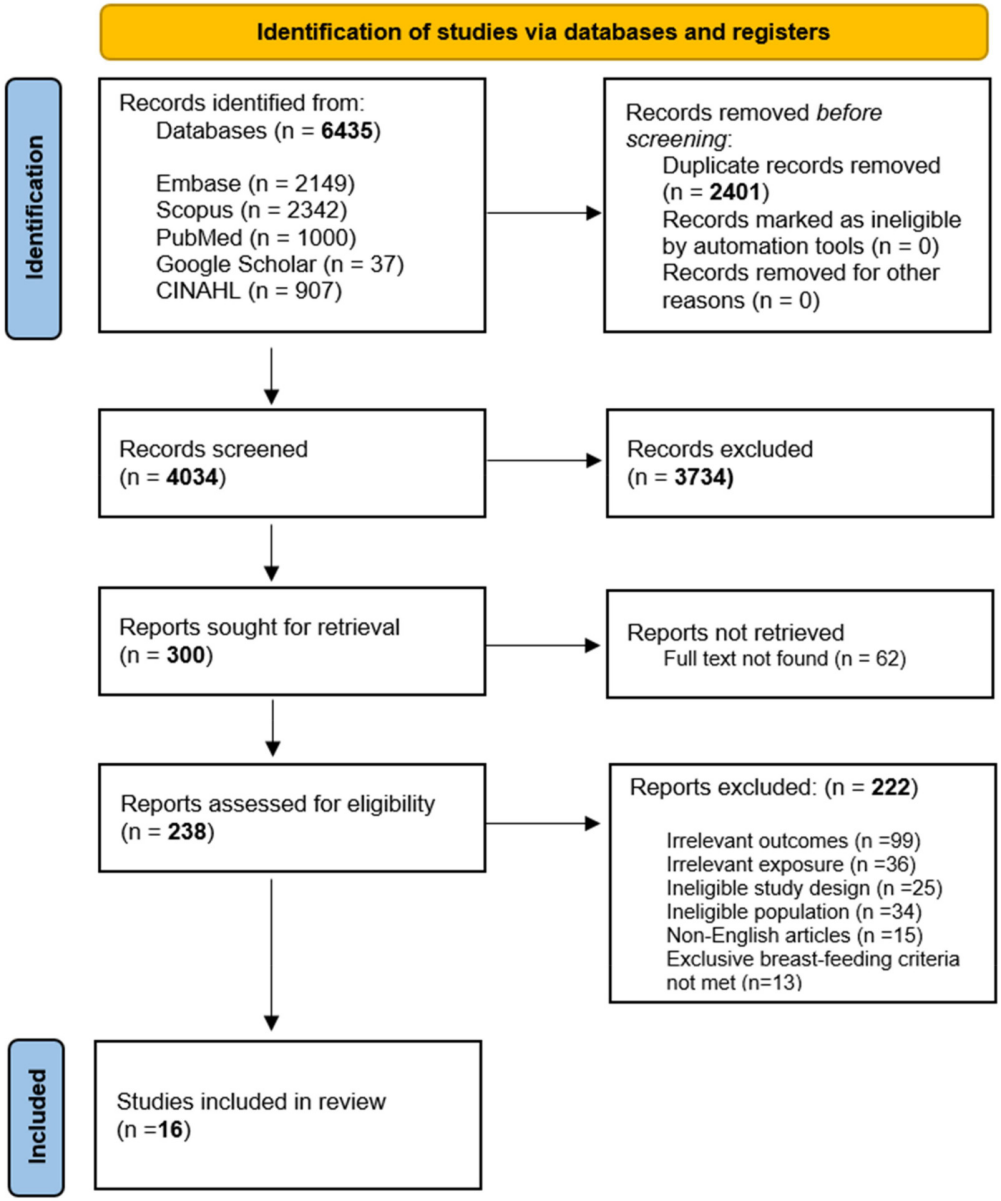
PRISMA flowchart depicting selection of studies.

The initial search across five databases yielded a total of 6,435 results. After eliminating 2,401 duplicates, 4,034 studies were screened based on their titles and abstracts. Following this, 238 full texts were assessed for eligibility, and 16 cohort studies were included in this systematic review (Figure 1).

### 3.1 Study characteristics

Thirteen (81.25%) out of 16 cohort studies included in this systematic review were conducted in high-income country settings. A total of 27,80,195 children were included in the selected studies, with sample sizes ranging between 715 and 16,56,682 participants. The risk of hospitalisation was assessed across various breastfeeding exposure statuses—EBF, PrBF, PBF and NBF. Outcomes were measured regarding odds ratios, incidence rate ratios, and relative risks. Risk of bias assessment showed that 12 (75%) out of 16 were rated as good quality, with four of them assessed to be of moderate quality studies due to low scores under categories such as selection of the non-exposed, non-comparability of the cohort based on designs, and inadequate duration of follow up (Table 2).

**Table 2:** Study characteristics.

SL No.	Author, year	Country	Setting	Sample size	Breastfeeding groups assessed	Age	Follow-up duration	Measure of outcome	Risk of bias
1	Yamakawa M et al., 2015	Japan	HIC	43,367	EBF, PBF	6 months	12 months	Odds Ratio	8*
2	Bahl R et al., 2005	Ghana, India, Peru	Peri-Urban, LMIC	9,424	NBF, EBF, PBF	18 to 24 days	6.2 Months	Rate Ratio	9*
3	Nakamura K et al., 2018	Japan	HIC	37,977	EBF, PBF	6 months	12 months	Odds Ratio	8*
4	Leung G et al., 2005	Hong Kong	HIC	6,608	EBF, PBF	3 Months	18 months	Odds Ratio	5*
5	Quigley M et al., 2016	England	HIC	18,818	EBF, PBF	<2months	9 months	Odds ratio	6*
6	César J et al., 1999	Brazil	LMIC	5,304	NBF, PBF	6–11 months	6 months	Odds Ratio	8*
7	Ladomenou F et al., 2010	Greece	HIC	6,878	NBF, PBF	1 month	6 months	Odds Ratio	5*
8	Quigley M et al., 2008	UK	HIC	18,819	NBF, EBF, PBF	1–8 months	9 Months	Monthly percentage risk	10*
9	Howie P et al., 1990	Scotland	HIC	750	NBF, EBF, PBF	0–13 weeks	24 months	Odds Ratio	5*
10	Lee J et al., 2023	South Korea	Rural, HIC	16,56,682	EBF, PBF	NA	NA	Incidence rate ratio	8*
11	Christensen N et al., 2019	Denmark	Urban, HIC	815	NBF, EBF, PBF	NA	2.8 (0.4)	Incidence rate ratio	8*
12	Sayed E et al., 1979	Canada	HIC	Na	EBF, PBF	NA	NA	Relative risk	9*
13	Nishimura T et al., 2009	Japan	Urban, HIC	892	EBF, TBF	NA	NA	0dds ratio	8*
14	Pature D C et al., 2019	France	HIC	18,329	NBF, PrBF, PBF	NA	NA	Odds ratio	10*
15	Kim J et al., 2021	South Korea	HIC	9,17,707	EBF, NBF	4–6 months	9 years	Risk Ratio	9*
16	Videholm S et al., 2021	Sweden	HIC	37,825	NBF, EBF, PBF	NA	NA	Incidence rate ratio	8*

EBF, exclusive breastfeeding; HIC, high-income countries; LMIC, low- & middle-income countries; NBF, never breastfed; PBF, partially breastfed; TBF, token breast feeding; PrBF, predominantly breastfed; risk of bias was assessed using new castle ottawa scale.

* Star scale rating.

### 3.2 Gastrointestinal infection-related hospitalisation

**Figure 2:**
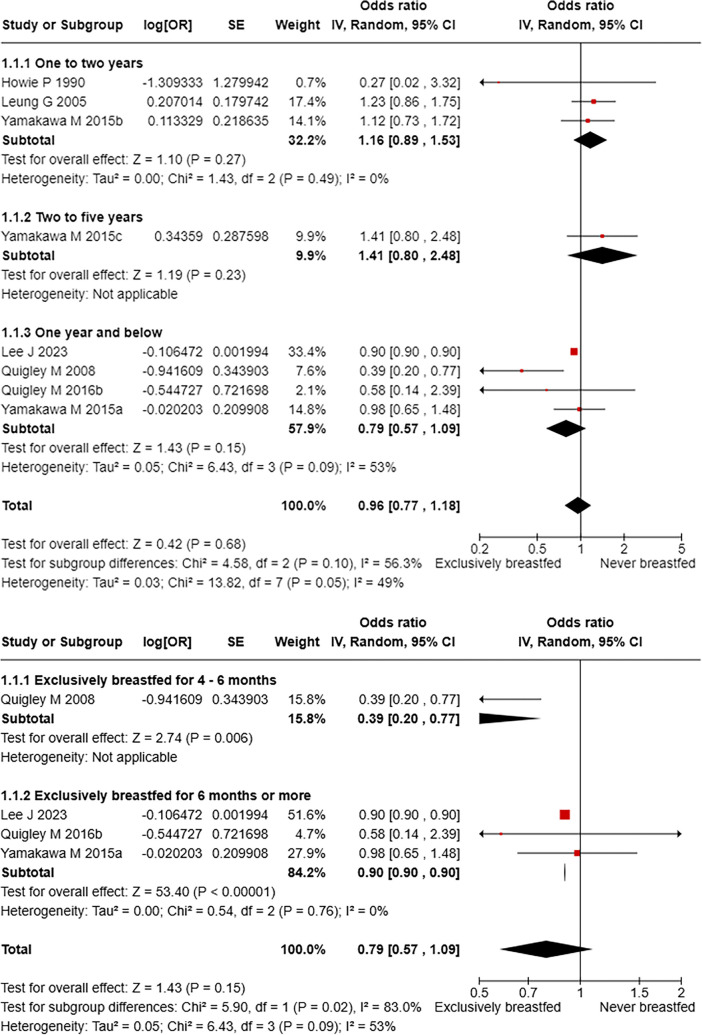
**(a)** Gastrointestinal infection-related hospitalisation among children exclusively breastfed compared to never breastfed. **(b)** Risk of gastrointestinal infection-related hospitalisation compared across different durations of exclusive breastfeeding under 1 year of age.

In total, six studies reported gastrointestinal infection-related hospitalisation among exclusively breastfed children compared to never breastfed. Pooled OR estimating chances of hospitalisation due to gastrointestinal infections in children under five years of age exclusively breastfed compared to those never breastfed is 0.96 (95% CI: 0.77–1.18) with a moderate heterogeneity (*I*^2^: 49%). While comparing the pooled estimates across the age groups, it is observed that the reduction in odds of hospitalisation due to gastrointestinal causes was greater in under 1-year children with exclusive breastfeeding, compared to those in 1–2 years and 2–5 years' age brackets (Figure 2a)*.* Furthermore, a meta-analysis comparing exclusive breastfeeding to predominant breastfeeding and partial breastfeeding showed that there is no significant reduction in hospitalisation with pooled ORs of 0.67 (95% CI: 0.23–1.98) and 0.45 (0.11–1.82), respectively (Table 3). About hospitalisations across different durations of EBF in <one year children, it was found that the protective effect of EBF duration of 4–6 months and six months or more have been comparable against hospitalisations, compared to never breastfed children (Figure 2b), however in similar comparison among children in 1–2 years' findings were non-significant (Supplementary Figure S1).

**Table 3:** Association between breastfeeding practices and gastrointestinal infection-related hospitalisation in children under five years of age.

Age group	Breastfeeding practices		Pooled odds ratio (95% CI)	No. of articles
	Exposure	Reference		
Exclusively breastfed vs. Never breastfed				
Overall	Exclusively breastfed	Never breastfed	0.96 (0.77–1.18)	6
One year and below			0.79 (0.57–1.09)	4
One to two years			1.16 (0.89–1.53)	3
Two to five years			1.41 (0.80–2.48)	1
Exclusively breastfed vs. Predominantly breastfed				
Overall	Exclusively breastfed	Predominantly breastfed	0.67 (0.23–1.98)	1
Exclusively breastfed vs. Partially breastfed				
Overall	Exclusively breastfed	Partially breastfed	0.45 (0.11–1.82)	1
Predominantly breastfed vs. Partially breastfed				
Overall	Predominantly breastfed	Partially breastfed	0.65 (0.35–1.20)	1
Predominantly breastfed vs. Never breastfed				
Overall	Predominantly breastfed	Never breastfed	0.63 (0.35–1.14)	2
One year and below			0.20 (0.08–0.50)	1
One to two years			0.86 (0.67–1.11)	1

### 3.3 Respiratory infection-related hospitalisation

**Figure 3:**
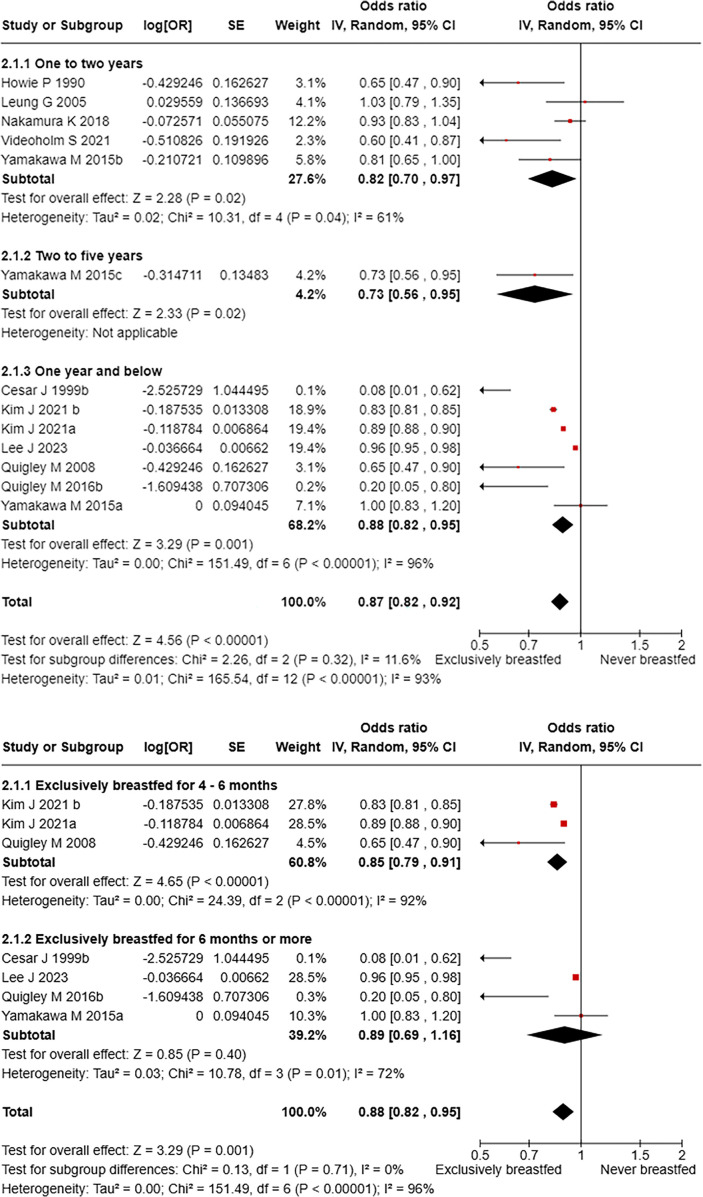
**(a)** Respiratory infection-related hospitalisation among children exclusively breastfed compared to never breastfed. **(b)** Risk of respiratory infection-related hospitalisation compared across different durations of exclusive breastfeeding in children under 1 year of age.

In total, 10 studies reported on respiratory infection-related hospitalisation among the under-five children compared the exclusively breastfed children with those who never breastfed. The pooled risk of hospitalisation (odds ratio) due to respiratory infections in children exclusively breastfed is 0.87 (95% CI: 0.82–0.92) when compared to never breastfed children with a substantial heterogeneity (*I*^2^: 93%). This significant protective effect of EBF against respiratory hospitalisation was consistent across age groups: <1 year, 1–2 and 2–5 years (Figure 3a). Further comparisons between breastfeeding practices showed that children receiving EBF significantly reduced the odds of hospitalisation by 0.53 (0.28–0.99) compared to those who were partially breastfed (Table 4). Comparisons of different duration of exclusive breastfeeding against never breastfed groups in children <1 year showed that EBF for four-six months had a significant protective effect against hospitalisation due to respiratory causes [OR: 0.85 (95% CI: 0.79–0.91)], while this association lost statistical significance when EBF of 6 months or more was considered to be the exposure (Figure 3b). In contrast, children between 1 and 2 years of age had a significant reduction in odds of hospitalisation among those who received EBF for six months and more [OR: 0.78 (95% CI: 0.64–0.95)] compared to never breastfed children (Supplementary Figure S2).

**Table 4:** Association between breastfeeding practices and respiratory infection-related hospitalisation in children under five years of age.

Age group	Breastfeeding practice		Pooled odds Ratio (95% CI)	No. of articles
	Exposure	Reference		
Exclusively breastfed vs. Never breastfed				
Overall	Exclusively breastfed	Never breastfed	0.87 (0.82–0.92)	10
One year and below			0.88 (0.82–0.95)	6
One to two years			0.82 (0.70–0.97)	5
Two to five years			0.73 (0.56–0.95)	1
Exclusively breastfed vs. Predominantly breastfed				
Overall	Exclusively breastfed	Predominantly breastfed	0.86 (0.45–1.64)	1
Exclusively breastfed vs. Partially breastfed				
Overall	Exclusively breastfed	Partially breastfed	0.53 (0.28–0.99)	3
Predominantly breastfed vs. Partially breastfed				
Overall	Predominantly breastfed	Partially breastfed	0.97 (0.59–1.60)	1
Predominantly breastfed vs. Never breastfed				
Overall	Predominantly breastfed	Never breastfed	0.84 (0.61–1.16)	2
One year and below			0.42 (0.16–1.09)	1
One to two years			0.93 (0.74–1.16)	1

### 3.4 All-cause and any infection-related hospitalisation

Two studies in total reported on the risk of all causes of infection-related hospitalisation among children under-five children, comparing exclusively breastfed children with those who never breastfed. The pooled reduction in odds of hospitalisation was estimated to be 0.97 (95% CI: 0.94–0.99) with lesser heterogeneity (*I*^2^: 16.2%). Similarly, a meta-analysis comparing predominantly breastfed children with those who never breastfed showed a significant reduction in the odds of all-cause hospitalisation [OR: 0.29 (95% CI: 0.15–0.57)]. Additionally, four studies have reported on any infection-related risk of hospitalisation among under-five children with a substantial heterogeneity (*I*^2^: 70%). Overall, a meta-analysis showed a 39% significant reduction in odds of infection-related hospitalisation among exclusively breastfed children [OR: 0.61 (95% CI: 0.37–0.99)] compared to those never breastfed. However, this borderline significance was lost in subgroup analyses based on age groups (Table 5). Comparisons of different durations of EBF against never breastfed children showed EBF for four-six months showed a significant protective effect against infection-related hospitalisation [OR: 0.52 (95% CI: 0.34–0.81)] (Supplementary Figure S3)*.*

**Table 5:** Association between breastfeeding practices and all-cause/any infection-related hospitalisation in children under five years of age.

Age group	Breastfeeding practice		Pooled odds Ratio (95% CI)	No. of articles
	Exposure	Reference		
All Cause hospitalisation				
Exclusively breastfed vs. Never breastfed				
Overall	Exclusively breastfed	Never breastfed	0.97 (0.94–0.99)	2
One year and below			0.97 (0.97–0.97)	1
One to two years			0.93 (0.86–1.00)	1
Exclusively breastfed vs. Predominantly breastfed				
Overall	Exclusively breastfed	Predominantly breastfed	0.77 (0.47–1.26)	1
Predominantly breastfed vs. Partially breastfed				
Overall	Predominantly breastfed	Partially breastfed	0.89 (0.62–1.28)	1
Predominantly breastfed vs. Never breastfed				
Overall	Predominantly breastfed	Never breastfed	0.29 (0.15–0.57)	1
Any infection-related hospitalisation				
Exclusively breastfed vs. Never breastfed				
Overall	Exclusively breastfed	Never breastfed	0.61 (0.37–0.99)	4
One year and below			0.68 (0.43–1.07)	3
Two to five years			0.25 (0.06–1.04)	1

Sensitivity analysis was not conducted in this systematic review due to the limited number of studies included in the age-group-wise meta-analysis, and also because these studies were rated to have low risk of bias. Since all meta-analyses models except one, included less than 10 number of studies, exploring publication bias was not attempted. Only one meta-analyses model i.e., Figure 3a was assessed for publication bias through funnel plot (Supplementary Figure S4).

## 4 Discussion

Our findings highlight the significance of duration and level of breastfeeding in the context of hospitalisation due to various causes. The summary is elicited in three primary sections: hospitalisation due to gastrointestinal causes, respiratory causes, and infections of any origin (viz., all infectious/any cause). Pooled estimates in each of the sections mentioned above suggested significantly lower odds of hospitalisation among the under-five children exclusively breastfed compared to the never breastfed ones except for the gastrointestinal cause, which is suggestive of the protective nature of exclusive breastfeeding.

In the first section, we observed a notable trend concerning gastrointestinal hospitalisations among children under five. Our comparison between exclusively breastfed children and those who were never breastfed suggests that exclusive breastfeeding (EBF) acts as a protective factor against gastrointestinal-related hospitalisations, aligning with findings from another study, thus providing a biological basis to the finding (15). Breastfeeding modulates the microbiota, which may induce regulatory T cells involved in T helper Th1/Th2 balance and enhance systemic innate immunity, explaining the protective effect of breastfeeding on subsequent hospital admission (16, 17). Though the findings were insignificant, they align with the findings of another SRMA, reporting a significantly higher risk of hospitalisation due to diarrhoea among not breastfed infants under five months of age [RR: 19.48 (6.04–62.87)] than those exclusively breastfed (18). Consistent with our findings, another study reported a significantly reduced risk of gastrointestinal infections in infants who were exclusively breastfed for six months or more (19). Further, a subgroup analysis based on age categories exhibited an increasing trend of odds with age, but the findings remain insignificant. It might suggest that as children age, the impact of breastfeeding may lessen as dietary diversity increases and they are exposed to more pathogens through external food sources (20). Another study revealed that breastfeeding, initiated within the first hour of birth, provided exclusively for six months and continued up to two years or beyond, improved child survival and well-being. Breastfeeding is considered to be the affordable, healthiest nutrition for the new-born child, having essential protective factors (21). When the under-five children predominantly breastfed were compared with those who never breastfed, it suggested significantly lower odds of hospitalisation, thus supporting that limited introduction of outside food to the children during early life prevents infection, thereby limiting the risk of hospitalisation (21).

In the second section of our study, we found a statistically significant reduction in the odds of hospitalisation due to respiratory infections among exclusively breastfed infants compared to those who were never breastfed or partially breastfed. And showed minimal variation when compared across the age groups. Supporting our findings, a systematic review reported that the risk of pneumonia-related mortality was significantly higher in infants aged 0–5 months who were not breastfed, with a relative risk (RR) of 14.97 (95% CI: 0.67–332.74). Additionally, for children aged 6–23 months, the risk of pneumonia mortality was 1.92 times higher in those not breastfed compared to their breastfed peers, although this result was less definitive (95% CI: 0.79–4.68) (22). In prior research, breastfeeding was found to significantly lower the risk of hospitalisation for respiratory infections, yielding a pooled relative risk of 0.43 (95% CI: 0.33; 0.55), with this advantage remaining consistent across various age groups. Comparisons between breastfed and non-breastfed children revealed an even stronger protective effect, with a pooled relative risk of 0.33 (95% CI: 0.24; 0.46). Moreover, breastfeeding was associated with reduced mortality from lower respiratory tract infections, reflected by a pooled relative risk of 0.30 (95% CI: 0.16; 0.56) (23). Next, comparing the duration of breastfeeding among children under one year depicted a higher protective effect among those breastfed for six or more months; though insignificant, it resonates with the findings of a study suggesting a decreasing trend in relative risk of hospitalisation as the duration of full breastfeeding increases in the first year of life (24). These findings align closely with our study, reinforcing the critical importance of breastfeeding in reducing hospitalisation and mortality associated with respiratory infections.

The third section pointed out that the odds of hospitalisation in both causes, viz., all causes/infection of any origin, were significantly lower among exclusively breastfed children under five than those who never breastfed. The pooled estimate showed no significant variation when compared across ages. A similar trend was seen when predominantly breastfed children under five were compared with those who never breastfed. This suggested that exclusive breastfeeding and predominantly breastfeeding acted as a protective factor for children under five against hospitalisation due to infectious causes. Our findings were consistent with previous research showing that the risk of infection-related mortality was significantly higher in infants who were predominantly, partially, or non-breastfed compared to those who were exclusively breastfed. In infants aged 0–5 months, the relative risks for predominantly, partially, and non-breastfed infants were 1.7, 4.56, and 8.66, respectively. For children aged 6–23 months, non-breastfed children had a two-fold increased risk of infection-related mortality compared to breastfed peers. These results highlight the protective effects of breastfeeding against infections and emphasise the importance of promoting optimal breastfeeding practices (25).

### 4.1 Strengths and limitations

This study has several advantages, including a comprehensive search of multiple databases, which ensures a systematic and comprehensive selection of relevant studies. Adhering to established PRISMA guidelines has enhanced the clarity and reliability of the findings. The inclusion of studies across the globe improves the generalizability of the results. However, there are some limitations, such as heterogeneity among the studies in terms of duration of exposure and outcome, which may complicate the interpretation of the overall findings and uncertainty of publication bias. Although this review included studies of cohort design that provide the advantage of establishing temporality between breastfeeding and hospitalization events, the limitation of having fewer studies in the meta-analyses is acknowledged in this review.

## 5 Conclusion

Our study underscores the critical role of exclusive and predominantly breastfeeding in reducing hospitalisation risks among children under five years of age across various health issues, including gastrointestinal and respiratory infections. The protective effects of breastfeeding were evident in significantly lower odds of hospitalisation and mortality, aligning with existing literature. Although some findings, particularly regarding gastrointestinal hospitalisation, were not statistically significant, they still reflect important trends. Nonetheless, these findings reinforce the need for public health initiatives that promote exclusive breastfeeding practices as an affordable and effective strategy for enhancing child health and preventing hospital admissions.

## Data Availability

The original contributions presented in the study are included in the article/Supplementary Material, further inquiries can be directed to the corresponding author/s.
