# Supplementary material for: Breastfeeding and risk of hospitalisation in children under five years—a systematic review and meta-analysis
Source: Front Pediatr. 2026 Feb 9;14:1748152. doi: 10.3389/fped.2026.1748152 (PMC12926449; doi:10.3389/fped.2026.1748152)
Supplement: Supplementary file 1 [file Table1.docx]

| PUBMED | | |
| --- | --- | --- |
| Search domain | **Search terms/strategy** | **No of hits** |
| P | (child) OR (children) OR (infant) OR (Pediatric)OR (Paediatric)  OR  (infant)OR(Toddlers)OR(low birthweight)OR(very low birth weight)OR(Neonate) OR (Preschool children) | 2,821,317 |
| E | [(breastfeeding) OR (breast feeding) OR (exclusive breastfeeding) OR (exclusive breast feeding) OR (breastfed) OR (breast fed) OR (breastfeeding,exclusive) OR (breast feeding,exclusive) OR(lactation)] **AND** [(duration) OR(time) OR (years) OR (months) OR(term) OR (weeks) OR (days) OR (period) OR (span)] | 65,647 |
| C | - |  |
| O | (hospitalis*) OR (hospitaliz*) OR (hospitalization admission) OR (nursing home) OR ( child,hospitalized) OR  (in patient) | 666,776 |
| S |  |  |
| P&E &O | [(child) OR (children) OR (infant) OR (Pediatric)OR (Paediatric)  OR  (infant)OR(Toddlers)OR(low birthweight)OR(very low birth weight)OR(Neonate) OR (Preschool children)]  **AND**  [[(breastfeeding) OR (breast feeding) OR (exclusive breastfeeding) OR (exclusive breast feeding) OR (breastfed) OR (breast fed) OR (breastfeeding,exclusive) OR (breast feeding,exclusive) OR(lactation)] AND [(duration) OR(time) OR (years) OR (months) OR(term) OR (weeks) OR (days) OR (period) OR (span)]]   **AND**   [(hospitalis*) OR (hospitaliz*) OR (hospitalization admission) OR (nursing home) OR ( child,hospitalized) OR  (in patient)] | 1000 |

**Search Strategy**

**Table 1: Search Strategy for PubMed**

| EMBASE | | |
| --- | --- | --- |
| Search domain | **Search terms/strategy** | **No of hits** |
| P | 'children'/exp OR children OR child:ab,ti OR pediatric:ab,ti OR infant:ab,ti OR toddler:ab,ti OR 'low birth weight':ab,ti OR 'very low birth weight':ab,ti OR newborn:ab,ti OR 'preschool children':ab,ti | 4,607,663 |
| E | 'breast feeding'/exp OR 'breast feeding' OR breastfeeding:ab,ti OR lactation:ab,ti OR 'breastfeeding, exclusive':ab,ti OR 'exclusive breast feeding':ab,ti OR breastfed:ab,ti OR 'breast fed':ab,ti **AND** 'duration'/exp OR duration OR time:ab,ti OR period:ab,ti OR months:ab,ti OR days:ab,ti OR weeks:ab,ti OR span:ab,ti | \| 70, 228 \| \| --- \| |
| C | - |  |
| O | 'hospitalization'/exp OR hospitalization OR 'child hospitalization':ab,ti OR 'nursing home':ab,ti OR 'hospital patient':ab,ti OR 'hospital admission':ab,ti OR hospitalisation:ab,ti | 767,344 |
| S |  |  |
| P&E &O | ['children'/exp OR children OR child:ab,ti OR pediatric:ab,ti OR infant:ab,ti OR toddler:ab,ti OR 'low birth weight':ab,ti OR 'very low birth weight':ab,ti OR newborn:ab,ti OR 'preschool children':ab,ti ]  **AND**  ['breast feeding'/exp OR 'breast feeding' OR breastfeeding:ab,ti OR lactation:ab,ti OR 'breastfeeding, exclusive':ab,ti OR 'exclusive breast feeding':ab,ti OR breastfed:ab,ti OR 'breast fed':ab,ti AND 'duration'/exp OR duration OR time:ab,ti OR period:ab,ti OR months:ab,ti OR days:ab,ti OR weeks:ab,ti OR span:ab,ti]**AND**[ 'hospitalization'/exp OR hospitalization OR 'child hospitalization':ab,ti OR 'nursing home':ab,ti OR 'hospital patient':ab,ti OR 'hospital admission':ab,ti OR hospitalisation:ab,ti ] | 2149 |

**Table 2: Search Strategy for EMBASE**

| SCOPUS | | |
| --- | --- | --- |
| Search domain | **Search terms/strategy** | **No of hits** |
| P | ( TITLE-ABS-KEY ( children ) OR TITLE-ABS-KEY ( child ) OR TITLE-ABS-KEY ( pediatric ) OR TITLE-ABS-KEY ( infant ) ) | 4,458766 |
| E | ( ( TITLE-ABS-KEY ( breastfeeding ) OR TITLE-ABS-KEY ( breast AND feeding ) OR TITLE-ABS-KEY ( lactation ) OR TITLE-ABS-KEY ( breastfed ) ) ) AND ( ( TITLE-ABS-KEY ( duration ) OR TITLE-ABS-KEY ( period ) OR TITLE-ABS-KEY ( month ) ) ) | 72471 |
| C | - |  |
| O | ( TITLE-ABS-KEY ( hospitalization ) OR TITLE-ABS-KEY ( hospitalisation ) OR TITLE-ABS-KEY ( 'nursing AND home' ) OR TITLE-ABS-KEY ( admission ) OR TITLE-ABS-KEY ( inpatient ) ) | 1182745 |
| S |  |  |
| P&E &O | ( ( TITLE-ABS-KEY ( children ) OR TITLE-ABS-KEY ( child ) OR TITLE-ABS-KEY ( pediatric ) OR TITLE-ABS-KEY ( infant ) ) ) AND ( ( ( TITLE-ABS-KEY ( breastfeeding ) OR TITLE-ABS-KEY ( breast AND feeding ) OR TITLE-ABS-KEY ( lactation ) OR TITLE-ABS-KEY ( breastfed ) ) ) AND ( ( TITLE-ABS-KEY ( duration ) OR TITLE-ABS-KEY ( period ) OR TITLE-ABS-KEY ( month ) ) ) ) AND ( ( TITLE-ABS-KEY ( hospitalization ) OR TITLE-ABS-KEY ( hospitalisation ) OR TITLE-ABS-KEY ( 'nursing AND home' ) OR TITLE-ABS-KEY ( admission ) OR TITLE-ABS-KEY ( inpatient ) ) ) AND ( EXCLUDE ( DOCTYPE , "ch" ) OR EXCLUDE ( DOCTYPE , "le" ) OR EXCLUDE ( DOCTYPE , "cp" ) OR EXCLUDE ( DOCTYPE , "no" ) OR EXCLUDE ( DOCTYPE , "sh" ) OR EXCLUDE ( DOCTYPE , "re" ) ) | 2342 |

**Table 3: Search Strategy for Scopus**
