# Supplementary figures and images for: Breastfeeding and risk of hospitalisation in children under five years—a systematic review and meta-analysis
Source: Front Pediatr. 2026 Feb 9;14:1748152. doi: 10.3389/fped.2026.1748152 (PMC12926449; doi:10.3389/fped.2026.1748152)

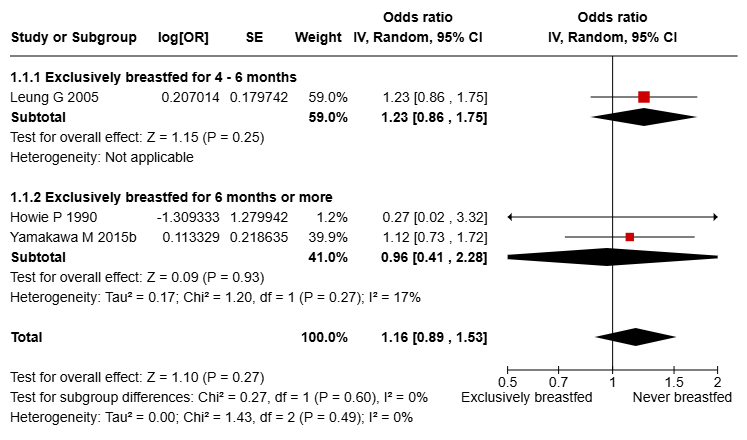

Supplement: Supplementary file 2 [file Image1.png]

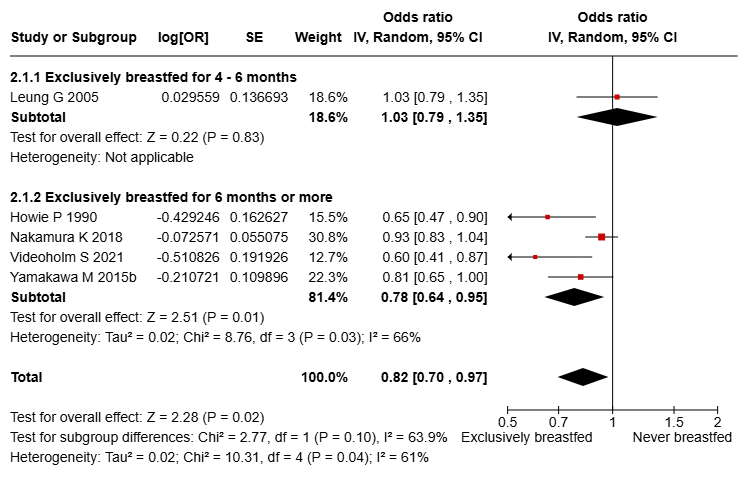

Supplement: Supplementary file 3 [file Image2.png]

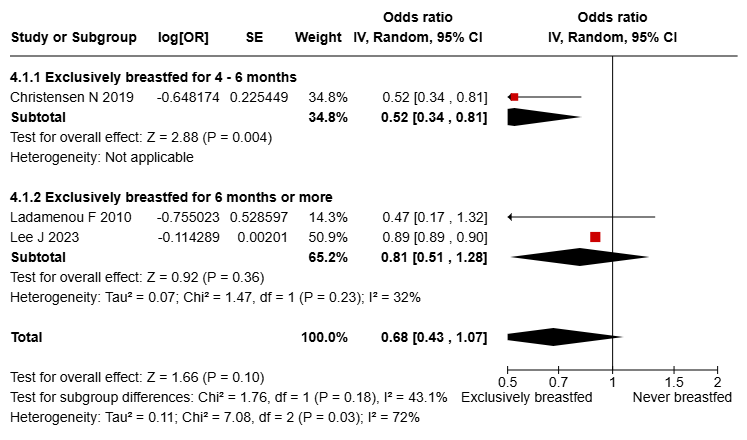

Supplement: Supplementary file 4 [file Image3.png]

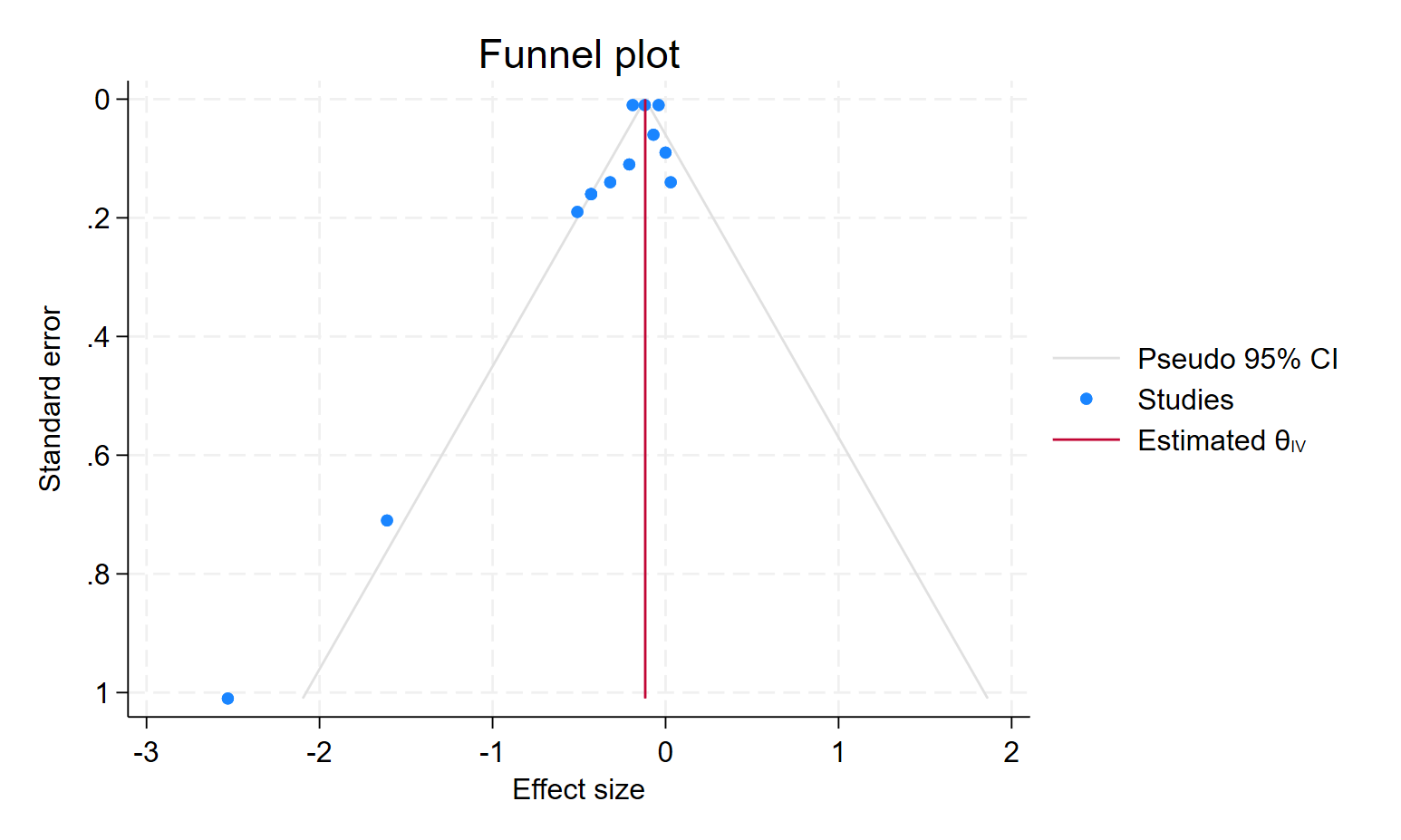

Supplement: Supplementary file 5 [file Image4.tif]
